# Shared decision making in designing new healthcare environments—time to begin improving quality

**DOI:** 10.1186/s12913-015-0782-7

**Published:** 2015-03-21

**Authors:** Marie Elf, Peter Fröst, Göran Lindahl, Helle Wijk

**Affiliations:** School of Education, Health and Social Studies, Dalarna University, Falun, Sweden; Department of Architecture, Chalmers University of Technology, Göteborg, Sweden; Department of Civil and Environmental Engineering, Chalmers University of Technology, Göteborg, Sweden; Sahlgrenska Academy, Health and Caring Sciences, University of Gothenburg, Göteborg, Sweden

**Keywords:** Design process, Healthcare architecture, Quality improvements, Shared decision-making, Person-centered

## Abstract

**Background:**

Successful implementation of new methods and models of healthcare to achieve better patient outcomes and safe, person-centered care is dependent on the physical environment of the healthcare architecture in which the healthcare is provided. Thus, decisions concerning healthcare architecture are critical because it affects people and work processes for many years and requires a long-term financial commitment from society. In this paper, we describe and suggest several strategies (critical factors) to promote shared-decision making when planning and designing new healthcare environments.

**Discussion:**

This paper discusses challenges and hindrances observed in the literature and from the authors extensive experiences in the field of planning and designing healthcare environments. An overview is presented of the challenges and new approaches for a process that involves the mutual exchange of knowledge among various stakeholders. Additionally, design approaches that balance the influence of specific and local requirements with general knowledge and evidence that should be encouraged are discussed.

**Summary:**

We suggest a shared-decision making and collaborative planning and design process between representatives from healthcare, construction sector and architecture based on evidence and end-users’ perspectives. If carefully and systematically applied, this approach will support and develop a framework for creating high quality healthcare environments.

## Background

Successful implementation of new methods and models of healthcare to achieve better patient outcomes and safe, person-centered care is dependent on the physical environment of the healthcare facility (or the healthcare architecture) in which the healthcare is provided [1]. Therefore, decisions concerning healthcare architecture are critical because such architecture affects people and work processes for many years and requires a long-term financial commitment from society [2,3]. Poor healthcare architecture can often lead to adverse events, such as patient falls, disorientation, healthcare-associated infections and patient dissatisfaction [4]. Additionally, poor healthcare architecture not only generates huge costs for society [5] but also decreases confidence in the healthcare system [6].

The design of healthcare architecture is thus an important quality factor in modern healthcare [7] and a healthcare organization must commit and integrate healthcare architecture into its strategic plans to improve the quality of care it offers [8,9]. Much of the enormous investment into new healthcare environments (HCEs) is neither based on the systematic acquisition of knowledge from practice or research nor seriously scrutinized by research-based follow-up procedures [10]. Similarly, there is only a limited amount of research available regarding the effects that HCEs have on matters such as healthcare efficiency, outcomes and safety compared with the amount of investment involved and the potential impact of HCEs on society [2,11].

### Person-centered care and person-centered architecture: the perfect match

Person-centered care considers both the physical and psychosocial aspects of an environment [12]. In 1861, Nightingale [13] described various factors in the physical HCE that promoted health and patient safety, including air quality, temperature, light and psychosocial features, such as views of nature and the proximity of staff and patients. Nightingale also pioneered the establishment of a direct link between healthcare architecture and patient health.

In the mid-1800s, hospitals were understood to have healing effects, and the buildings were frequently situated in park-like settings with abundant greenery. Designing hospitals and healthcare processes involved the holistic consideration of the entire, undivided environment. In the mid-1900s, a functionalist design philosophy emerged, and designing healthcare buildings reflected the era's technical and rational approach to healthcare [14]. The structure of healthcare organizations was inspired by a by a rationalistic and production based ways of thinking inspired by scientific management and methods from industry [15] developed for the industry that included separated units for various medical specialties often categorized based on medical diagnosis with the risk of an objectification of the patient. The pathological perspective divides humans into dysfunctional “parts” needed to be repaired. The approach considers health to be the opposite of diseases and effective treatment to be carries out as an assembly line. This approach influenced and placed demands on the design of HCEs, which manifested rationality in an infinitely expandable structure. Patient experiences were mostly ignored. Many healthcare facilities that we think of as “not supportive” evolved from this approach to design [16].

Currently, there are several reasons for the rebirth of a person-centered approach to designing the physical HCE. Based on research and articulated as evidence-based design (EBD) [17,18], it has been demonstrated that the physical HCE promotes or influences health, reduces the duration of treatment, decreases medication requirements and helps reduce the stress experienced by patients, their families and the teams caring for them [4]. Thus, innovations in healthcare architecture are an important part of the healing process itself instead of a functional three-dimensional structure in which care is provided. Additionally, patients demand safe, welcoming and attractive environments that can support them and their families when they are affected by illness [6]. Therefore, the concept of person-centered care is a priority for both healthcare and healthcare architecture. Structures and methods that are developed to ensure interaction among a variety of disciplines should be implemented to create new HCEs [9,19].

### The initial planning and design phase

The most critical decisions in the planning and design process (PDP) are made in the early phases [20,21]. The initial phase is typically characterized as a conceptual phase in which stakeholders meet to discuss ideas and space requirements and prepare for design decisions [22-24]. This phase is also when new innovative care models are integrated with building design development, spatial issues and various perspectives [9]. The primary purpose of this phase is to define the HCE from the user’s perspective and relate this perspective to the healthcare organization’s strategic plan [8,25]. User needs must be identified and properly articulated because they will now be analyzed based on external factors, such as new healthcare models and requirements, the specific context and available resources [26]. Therefore, planning the HCE requires a thorough analysis of patient-related objectives that the healthcare facility is expected to meet and an integration of the processes (care activities) and spatial conditions necessary to achieve such objectives [24,27-29]. Clearly, analyzing objectives must be a shared responsibility that is implemented in collaboration with stakeholders representing patient groups, healthcare staff, architects and engineers, facility organization members and, if possible, contractors. The decisions must be informed by the various perspectives and must be carried through to the planning and design steps and to the subsequent production process. The strategic involvement of healthcare professionals in the PDP is essential for integrating knowledge of the care processes into the architectural design. Whenever feasible, representatives from groups including patients, families and consultants should also participate [30,31]. This initial phase greatly influences the building project and its end results; therefore, it also impacts the organization’s ability to control future healthcare outcomes [32]. A poorly conducted PDP can lead to low quality project outcomes, dissatisfaction among users, delays and higher costs [11,23].

In this paper, we describe and suggest several strategies (critical factors) to promote shared-decision making when planning and designing new HCEs. If carefully and systematically applied, this approach will support and develop a framework for creating high quality HCEs.

## Discussion

### Starting point for change

Historically, there has been an interest in the impact of the HCE on people’s health and wellbeing in healthcare facilities (e.g., the HCE is a key concept in nursing). However, the effect of the HCE on the quality of life and care has only recently been systematically explored and analyzed. There is a growing focus on the HCE and its relationship to health and wellbeing for various patient groups, which has resulted in the development of more effective PDPs. New research centers are being established around the world to assemble the best researchers in the field. Conferences join people together from the fields of architecture and healthcare, and joint research projects are being undertaken by students of architecture and healthcare who are studying the same research question from different perspectives. With grants from research funds such as the, the Swedish Healthcare facilities network [33] and the Swedish Research Council Formas [34], Center for healthcare architecture (CVA) at Chalmers University of Technology in Sweden has been launched [35] a center that aims to coordinate and implement research and development on the relationship between healthcare and architecture. This cross-disciplinary center aims to contribute and develop knowledge and methods that can bridge the boundaries between the disciplines. Despite these developments, methods and strategies that safeguard quality in planning and design practice must still be established. Efforts to direct the PDP toward a more collaborative and evidence-based approach are receiving increased attention.

### Challenges

One of the challenges of healthcare architecture is to truly integrate the needs of future users into design decisions [24]. Additionally, design approaches that balance the influence of specific and local requirements with general knowledge and evidence should be encouraged [36-38]. Moreover, even when the design of the environment is acknowledged as an important part of the overall quality of healthcare, the characteristics, issues and policies of the design are frequently not included in the organizational strategic plans.

Many healthcare professionals want to contribute and influence the design process but do not understand how, why and in what way they can be involved, which is similar to architects and planners from their vantage points on the other side of the process. Therefore, the challenge is to generate a collaborative and learning process that focuses on end-user perspectives and utilizes evidence-based knowledge. This method stands in sharp contrast to construction meetings that focus primarily on room size and building location.

### Critical factors for ensuring the quality of HCE

A new approach to the PDP has been appropriated from research and practice that requires healthcare and construction and design to utilize a more collaborative and evidence-based design process to secure efficiency and effectiveness. However, this design approach must be applied systematically and consistently. We therefore propose a collaboration-based framework to facilitate this change and establish design outcomes. We suggest the following critical factors for a quality-driven PDP:

1. Collaboration for shared decisions.
2. Integration of evidence and experiences.
3. Focus on outcomes.
4. Use of documentation.
5. Evaluation of healthcare environments.

### Collaboration for shared decisions

The importance of collaboration cannot be overemphasized when fostering ownership of and participation in design decisions by users. Collaboration is essential to designing architecture that will complement current and future healthcare processes [39-41]. Currently, the concept of co-design has been established to express the importance of both co-operation and creation in the PDP [42,43].

A true collaborative process in this regard is characterized by design planning and learning and is a context in which the problem is defined during the process. The work is collaborative and interactive, and the goals of the project are redefined regularly in parallel as new conditions emerge that further define the process and as new knowledge emerges from the shared process [42]. This type of process is more decentralized than earlier processes (i.e., there are fewer standardized frameworks or design solutions transferred to individual building projects). Thus, knowledge and innovation emerge locally through individual projects.

An advantage of this collaborative planning model is that it allows the healthcare organizations’ specific needs and wishes to be integrated into the process. A weakness of this approach is that individual projects may be too focused on current needs and requirements [44] and may not fully use and implement available research and best practices. There is a risk that “the wheel will be reinvented” both now and in the near-term future. The effective use of the dynamic model therefore requires the support of systematic knowledge transfer from practice and research.

### Integration of evidence and experience

There are several examples of successful collaborative projects involving patients, staff, architects and planners [39,40,44]. One example, based on design process thinking, is called “Design dialogues”, a method with a collaborative approach developed at Chalmers University of Technology in Sweden [40,44] to address the need to integrate user perspectives to reach innovative solutions in the PDP. The method has been tested and found to improve the design outcome. Another method, used and tested in PDP is using modelling and simulation with system dynamics as a communication tool [39]. However, these projects rarely describe how the physical environment affects health and well-being or the healthcare process itself. Therefore, evidence-based design (EBD) has been introduced in planning and design to inform and guide stakeholders and decision-makers regarding what approaches are most likely to work and be successful over the long run [18].

### Evidence-based design

EBD is a recognized method of ensuring the quality of HCEs [45], although the method remains theoretical and has not been fully implemented in practice. In addition, the quality of the research in the area is still needed of a rigorous appraisal [46]. EBD can be defined as a critical and reflective process in which decisions about the building environment are based on credible research results, evaluations and systematically analyzed experience from existing environments that consider the user experience, in particular [17,18,29]. Although EBD assumes that decisions about the building environment should be based on evidence regarding the effects of different design solutions on people, organizations and other factors (including costs), such evidence must also include relevant research regarding the needs of those people who will utilize the physical environment being constructed. The EBD must also consider and include approaches and concepts such as person-centered care, participation in care and teamwork.

EBD is closely related to other methods of continuous quality improvement [10,47,48]. The concept requires that the objectives of the HCE be defined by the best available research, knowledge and experience—in addition to explicit measures of the project’s expected outcomes—to enable evaluation after the building has been completed and is in use. An EBD process must be informed by multiple perspectives, including those of healthcare professionals, architects and engineers, and should be communicated via briefs and programs developed during the PDP.

### Focus on outcomes

There are several reasons to emphasize the importance of outcomes and the measurement of outcomes during the PDP. It is foremost patient related outcomes measures that needs to be defined in beforehand, i.e. outcomes expected to be achieved by an adapted environment that is of value for the patient [49]. Outcomes that are based on quality of care, health status, processes and the patient experience [50]. A focus on outcomes directs attention toward the main goals of the new environment, and users are the central focus of the planning and design; thus, design decisions should be based on the results of these outcomes. This approach also forms the basis for finding and evaluating evidence concerning the needs of future users of the environment and the influence and impact of various design solutions on individual health. At present, however, spatial issues, such as the number of rooms and square meters, typically remain the focus during traditional early planning stages rather than trying to correlate users’ needs and outcomes. Research has also revealed deficiencies in the quality of the planning process with respect to focusing on the needs of users (person-orientation) and clarifying patient-related outcome measures for subsequent evaluation of the actual building or facility [29,47,51,52].

### Using documentation

Design decisions should be conveyed through documents (design proposals, programs and/or briefs) developed in the PDP. These documents are key for decision-making in a building process [53]. The primary aim of recording information in a document is to support the forthcoming building process and the related decision-making process and to facilitate communication between the stakeholders involved [54]. Additionally, documentation is a valuable source of quality assurance because it ensures the transparency of the process and the effective evaluation of the completed building.

The initial program relates to the strategic plan of the organization (i.e., the healthcare organization) and describes how the design of the building contributes to the organization’s established strategic goals [8,55]. Additionally, tangible performance measures, written documents and evidence-based information in the planning process are increasingly required.

Although a poor end result of the program may indicate that the planning process was low quality, few authors have attempted to review the information in the programs. Elf and Malmqvist [51] conducted a review of the information in programs created for the HCE planning process and showed that only a few programs had explicit patient-focused goals for the project, measurable outcomes or references to new evidence. Fewer than half of the programs reviewed involved a clear description of the organization’s objectives and the activities that would occur in the planned care environment. A recent study of the content and quality of programs found the same pattern [52].

### Evaluation of healthcare settings

An evaluation of HCEs is important to gather knowledge and experience about how the environment works and how it is perceived by the people using it. Evaluations are a prerequisite for successful and continuous quality improvement of the PDP and of the healthcare architecture [56]. Evaluations of the completed environment are seldom conducted, and even rarer is it to already in the PDP plan for an evaluation. This may be related to the ambiguity of whose responsibility it is to evaluate a completed building and who should bear the cost for such evaluations. In addition, the lack of evaluations may be due to that the planning process is project based by practitioners only taking responsibility for their own learning, a situation without a tradition of continuous improvements approach. There may also be deficiencies of knowledge and skills about how valid assessments should be performed and also what should be evaluated.

One method to evaluate a building and to utilize users’ perspectives regarding its functionality is to conduct a post-occupancy evaluation (POE) [57], which is an examination of a completed room or entire building. POE includes a systematic description of users’ impressions and behaviors in the building. The goal of a POE is to assess how the business is related to the design and how the design is perceived by users. The POE also aims to generate knowledge that can be considered in the planning of new facilities or buildings.

A critique of POEs is that they have focused on experiences and opinions of users with regard to buildings and functions rather than on evaluations of predetermined quality criteria using a validated instrument to measure the quality of the building design [47]. Another criticism is that POE has been conducted as research instead of as a natural extension of the PDP to ensure continuous improvement [47].

Another approach that has broader applications to this process is the building performance evaluation (BPE) method [58], which is described as a continuous process that systematically evaluates building performance and efficiency according to documented criteria. These criteria should be based on evaluations of existing environments, evidence-based knowledge and innovative techniques for designing and constructing for new buildings [55].

Evaluation methods are critically important because explicit goals are operationalized and presented at the beginning of the PDP to enable evaluation after the building is completed and being used.

## Summary

A collaborative PDP that is based on integrating evidence and end-users’ perspectives will have a profound impact on the quality of healthcare architecture and thus patient health and quality of care. This paper has described how the design of new healthcare architecture is a process that involves the mutual exchange of knowledge among various stakeholders. The healthcare organization is affected by the design of the HCE, but the environment is also affected by the organizational dynamics of the specific healthcare organization involved.

A potential difficulty results if the PDP is considered merely the ‘diffusion’ of an idea or the “rolling out” of a fixed drawing from the planners or architects. However, if PDP is considered a process of organizational change and development, it can be used strategically to transform the organization, and the design can be integrated and continuously improved. We believe that such a process can only occur when properly supported by both evidence and the needs of the users. An evidence-based framework for the PDP is crucial; such a framework enables ‘user needs’ to be articulated. In establishing this framework, user input can become a coherent, steering force that transforms and specifies the overall vision to create a solid basis for the organizational transformation envisioned.

Finally, the planning and design of new healthcare architecture is a careful balance among various dynamics and jointly developed and defined goals for the project without pre-specifying or controlling the details of the process. This process is thus a balancing act between setting goals for the project and encouraging a mutual learning process that will inevitably change those objectives. Accepting and taking advantage of this indisputable uncertainty might be the most difficult lesson to learn. The most successful PDPs are those in which a commitment to management and planning occurs simultaneously with experimentation and mutual learning.

These insights are essential for a successful PDP. Nevertheless, these suggestions should not be viewed as a definitive list of critical success factors or as a recipe that will guarantee the avoidance of mistakes. Actual attempts to formulate such a list are contrary to the essential thrust of the process described herein.

In the end, a successful PDP can only be identified by actual achievements and outcomes that are measured and that become a part of experience and evidence. It is therefore necessary to couple creativity, innovation and evidence with multi-disciplinary teams to realize the full potential of shared decision-making regarding healthcare architecture.

## Abbreviations

HCE: Healthcare environments
PDP: Planning and design process
